# Levels of Processing Effects on Memory for Color-Object Associations

**DOI:** 10.5334/joc.437

**Published:** 2025-03-04

**Authors:** Mirela Dubravac, Chhavi Sachdeva, Nicolas Rothen

**Affiliations:** 1 Faculty of Psychology, UniDistance Suisse, Brig, Switzerland

**Keywords:** levels of processing, color recall, color-object association, memory

## Abstract

The levels of processing effect demonstrates that deeper encoding (processing meaning) enhances memory retention more than shallow encoding (processing perceptual features). While extensively studied with verbal materials, limited research has addressed this effect using nonverbal materials such as pictures. Previous studies have used pleasantness judgments to induce deep encoding and judgments of straight lines to induce shallow encoding. However, these tasks confound level of processing with other factors like task relevance, self-reference, and attentional scope, offering alternative explanations for observed memory performance differences. This online study (*N* = 307) tested the levels of processing framework for pictures using novel encoding tasks to isolate the effect of semantic processing. The novel encoding tasks involved a size judgment. In the shallow encoding condition, participants compared the size of an object to the displayed size of a reference object presented on the screen. This is a perceptual comparison of two pictures. In the deep encoding condition, participants compared the size of an object to the real-life size of a reference object. This requires an understanding of the semantic meaning of the presented object. Our results showed better memory performance in deep encoding conditions (pleasantness judgment, real-life size judgment) than shallow encoding conditions (line judgment, displayed size judgment), supporting the levels of processing framework. Additionally, the new size judgment tasks minimized alternative explanatory factors, providing a clearer understanding of how semantic processing influences visual associative memory. These findings confirm the applicability of the levels of processing effect to nonverbal materials.

Typically, people remember more words when they process their meaning (e.g., judging animacy) rather than their perceptual features (e.g., judging letter size). This phenomenon, known as the levels of processing effect, highlights the difference between deep and shallow encoding conditions (Craik & Lockhart, 1972). Many studies have replicated this effect using verbal materials, such as words (Craik & Tulving, 1975; Leding, 2018; Rhodes & Anastasi, 2000). Only a limited number of studies have also investigated the effect using nonverbal materials, such as pictures (Baddeley & Hitch, 2017; Bower & Karlin, 1974; Ovalle-Fresa, Uslu, et al., 2021). These studies typically induce deep encoding through pleasantness judgments and shallow encoding through other types of judgments, such as determining whether a picture contains a straight line. However, the pleasantness judgment can be problematic as it allows for alternative explanations for improved memory performance. In the present study, we addressed this issue by using alternative judgments to induce deep and shallow encoding, providing a more direct test of the levels of processing framework for pictures.

We build on work by Ovalle-Fresa, Uslu, et al. (2021), who investigated the levels of processing effect for object-color associations. Specifically, we replicated their Experiment 3, in which participants encoded the colors of objects for later recall. Participants encoded the object-color associations under either deep or shallow encoding conditions. In the deep encoding condition, participants indicated whether the object was pleasant for them, while in the shallow encoding condition, they judged whether there was a straight line in the object. Following each encoding block, participants completed a recall block, setting the recalled color for each object using a color wheel. Results showed that the set color was consistently closer to the target color in the deep encoding condition, indicating better memory. The authors concluded that the levels of processing framework also applies to basic perceptual features (i.e., color) in visual associative memory.

To strengthen their conclusions, we conducted a study to replicate their results in an online setting and extend their study by using alternative instructions for the deep and shallow encoding conditions. The rationale for using alternative instructions is that the pleasantness judgment and the straight-line judgment differ not only in the level of processing but also in other aspects such as task relevance of the color, self-reference, and attentional scope. This is problematic because it allows for alternative explanations for the observed differences in memory performance. Next, we will outline three alternative explanatory accounts for levels of processing manipulations with pleasantness judgments.

When asked to judge an object’s pleasantness, wouldn’t you naturally consider its color? Conversely, how relevant is color when determining the presence of a straight line? Intuitively, color likely influences pleasantness judgments more than assessments of line straightness. Thus, color seems to be more relevant to the pleasantness judgment than to the straight-line judgment. Given that the memory test involved recalling the color, the task-relevance of color may have enhanced memory performance in the pleasantness judgment condition. This assumption is supported by research demonstrating better memory for task-relevant information compared to task-irrelevant information (Dubravac & Meier, 2023; Richter & Yeung, 2012). Therefore, the task relevance of color could offer an alternative explanation for differences in memory performance.

The two judgment conditions also differ in their relevance to the self. Pleasantness judgments, whether intentionally or unintentionally, often draw on prior experiences with similar objects, as well as our attitudes towards the objects. This process triggers personal associations and connects the judgment to us. Conversely, assessing a picture for the presence of a straight line rarely involves such self-referential processing. We propose that the self-reference component of the pleasantness judgment could enhance memory. This assumption is supported by research demonstrating better memory for information that is personally relevant (Levine & Edelstein, 2009; Rogers et al., 1977; Symons & Johnson, 1997). Therefore, self-reference could offer an alternative explanation for differences in memory performance.

Another difference worth discussing in the context of memory performance is attentional scope. Pleasantness judgments typically elicit a broader, more global attentional scope, encompassing the object as a whole. In contrast, straight-line judgments require a narrower, more focused attentional scope, targeting specific parts of a picture. Thus, the two judgment tasks differ in the attentional scope required for task completion. At recall, participants are presented with the entire object and asked to recall its color. Here, the attentional scope is presumably broader, as focusing on a specific part of the picture would not aid in recalling the overall color of the object. Therefore, the more consistent attentional scope between encoding and recall in the pleasantness judgment task may better support memory recall in this condition (cf. Morris et al., 1977). We argue that the broader attentional scope in the pleasantness judgment task may enhance color memory compared to the narrower attentional scope in the straight-line judgment task. This assumption is supported by research demonstrating better memory for holistically encoded information (Bower & Karlin, 1974; Muhmenthaler et al., 2023). Therefore, attentional scope could provide an alternative explanation for the differences in memory performance.

To avoid these alternative explanations, we searched for two conditions differing exclusively in the levels of processing. We used a size judgment task, where participants judged the size of a colored object relative to a displayed reference object (i.e., a shoebox). In the deep condition, participants compared the real-life size of the colored object to the size of the reference object. In the shallow condition, participants compared the displayed size of the two objects on the screen. In other words, the deep encoding condition entailed a real-life size judgment, while the shallow encoding condition entailed a displayed size judgment. The critical difference between conditions lies in the need for semantic processing of the colored object. In the deep condition, participants had to understand the semantic meaning of the object to determine whether it is bigger or smaller than a shoebox. In the shallow condition, it was a purely perceptual decision, with no need to process the object’s semantic meaning. These conditions provide a purer manipulation of the levels of processing.

Summarizing, the aim of the present study was to provide a more direct test of the levels of processing framework for pictures by using novel encoding instructions designed to prevent alternative explanations, thereby addressing a limitation found in previous studies (Baddeley & Hitch, 2017; Ovalle-Fresa, Uslu, et al., 2021). To this end, we replicated and extended the study by Ovalle-Fresa, Uslu, et al. (2021). We manipulated the levels of processing (deep vs. shallow) with two types of instructions: original pleasantness/line judgment versus new real-life/displayed size judgment.

If the levels of processing framework applies to basic visual perceptual features (i.e., color) in visual associative memory, memory should be better in deep encoding conditions (pleasantness judgment, real-life size judgment) than in shallow encoding conditions (line judgment, displayed size judgment). In our design, this would be reflected in a main effect of levels of processing. If alternative explanations are (also) responsible for the effect observed in the original study (Ovalle-Fresa, Uslu, et al., 2021) and related studies (Baddeley & Hitch, 2017), we expect to observe an interaction. Specifically, we expect better performance in the pleasantness condition compared to the line judgment condition, but a reduced performance difference between real-life size judgment and displayed size judgment conditions. The study was preregistered on OSF (https://osf.io/enmp2).

## Method

### Participants

Our aim was to achieve a sample size that would provide sufficient power, and we planned not to recruit additional participants if the minimum power criterion of .80 was met. To detect a potential interaction in a 2 × 2 split plot design for a minimal effect of interest (Cohen’s *d* = .4) at the standard .05 alpha error probability with .80 power, a minimum of 200 participants is required (Brysbaert, 2019). Each student in a course on scientific practice and writing was tasked with recruiting 16 participants. Students in the course recruited a total of 341^1^ participants by word of mouth.

We obtained complete data of 317 participants. Based on the preregistered criteria, we excluded two participants because they indicated to be older than 65. Further, we excluded eight participants who indicated in our post-test questionnaire that they did not follow instructions. The final sample consisted of 307 participants (158 in the replication and 149 in the extension instruction condition). Based on the responses on the demographics questionnaire, 170 participants were women, 132 were men, and 5 preferred not to disclose their gender. Their ages ranged from 18 to 64 years old (*M* = 38 years, *SD* = 11 years). Seven participants identified as ambidextrous, 35 as left-handed, and 265 as right-handed. All participants reported the required level of German proficiency.

The study procedure adhered to ethical guidelines in accordance with the Declaration of Helsinki and was approved by the local ethics committee (#2024-01-00002).

### Material

Stimuli consisted of 160 unique pictures of objects taken from the BOSS database (Brodeur et al., 2010). Using the R package *magick* (Ooms, 2023; version 4.8.1) the pictures were converted from jpg to png format and were then subsequently converted to grayscale. Using the same package, the pictures were then given a transparent background and adjusted to red with an opacity of approximately 49. Subsequently, we calculated the number of transparent pixels around each picture to determine their size. Then, any picture consisting of over 120,000 transparent pixels were removed due to their limited surface for color determination. The pictures were displayed in one of 80 colors, which were approximately equidistantly sampled on a color wheel. A picture of a shoebox retrieved from the web (https://www.pngwing.com/en/free-png-mcqyf) served as a reference picture for the size judgment tasks.

The 160 objects were divided into two sets of 80 stimuli each, which were further assigned to 10 lists of eight stimuli each. Similarly, the 80 colors, sampled^2^ around a HSL (hue, saturation, lightness) color wheel (Sachdeva et al., 2024), were assigned to 10 lists of eight colors each. The colors within each list differed by 45° on the color wheel. The color lists were pseudorandomly assigned to the stimulus lists. The lists appeared in random order. Within these lists, the order of colors and the order of stimuli were randomized separately, to ensure a random stimulus-color association. Also, within these lists, half of the trials displayed the reference picture larger (1.5×) than the colored object, and the other half displayed the reference picture smaller (0.5x). This order was also randomized within lists. In the deep condition, half of the selected objects were likely smaller than a shoebox in real life, while the other half were likely larger.

The same 80 colors were used for both conditions (deep & shallow). The assignment of the stimulus sets to the conditions was counterbalanced across participants. The order of conditions and therefore also the order of set presentation was also counterbalanced across participants. Stimulus presentation was controlled with lab.js, an online experiment builder (Henninger et al., 2022).

### Procedure

Participants received the link to the online study via email. After reading the general study information and providing informed consent by clicking on the respective button, participants first completed a display calibration procedure. Participants set the size of a window to match the size of a credit card by moving a slider. Next, they completed an Ishihara color blindness test (Clark, 1924) with 20 pictures presented in random order. If they gave less than 70% correct responses a message appeared informing participants that the following task may be frustrating to them if they have trouble with distinguishing colors. They could still take part in the study if they wished. Otherwise, the procedure continued with the instructions for the main part of the study.

Half of the participants received the replication instructions (pleasantness vs. line judgment) and the other half received the extension instructions (real-life vs. displayed size judgment). Besides the instructions the only other difference was that the participants with the extension instructions saw the colored object along with the reference picture, which varied in size in both deep and shallow conditions. Thus, the stimuli, display and procedure were the same in both deep and shallow condition. The only difference was the instructions. The extension instruction was to indicate whether the object was bigger (by pressing the key “g”) or smaller (by pressing the key “k”) compared to a shoebox in real-life (deep encoding condition) or compared to the shoebox displayed on the screen (shallow encoding condition). The replication instruction was to indicate whether they liked the object (by pressing the key “g”) or not (by pressing the key “k”; deep encoding condition) or whether the object contained a straight line (by pressing the key “g”) or not (by pressing the key “k”; shallow encoding condition).

The order of encoding conditions was counterbalanced across participants. Each condition consisted of instructions with example pictures and a practice block followed by 10 experimental blocks. Each block consisted of eight encoding trials immediately followed by eight recall trials. During encoding, participants were instructed to memorize the object-color associations for a subsequent memory test. The order of object presentation during encoding and recognition was randomized for each participant.

Each encoding trial started with a central fixation cross (1s), followed by the stimulus display (1s), which involved a single-colored object in the middle of the screen (replication instructions) or a colored object in the middle with a shoebox on the right side (extension instructions). Next, the respective instruction was displayed on the screen (2s) prompting the response. After 2s the next trial started. If participants did not respond within 2s it was counted as a missed response. After eight encoding trials started the recall phase.

**Figure 1:**
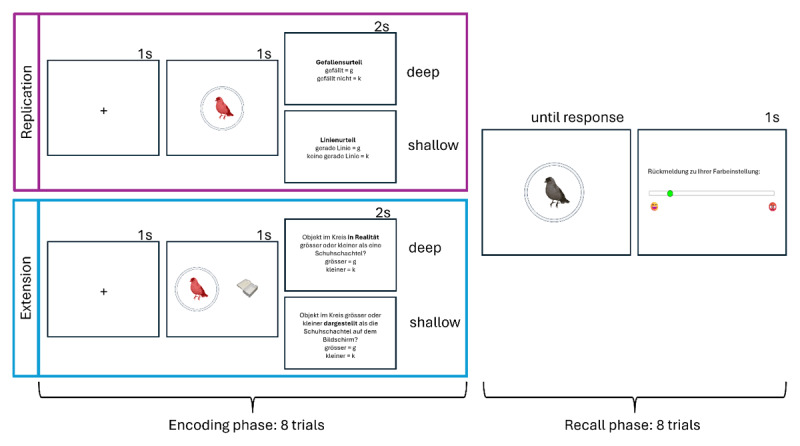
Example Encoding and Recall Trial. *Note*. The encoding phase involved presenting the colored object in the middle of the screen (replication) or next to a reference object (extension condition) followed by a response prompt. In the replication condition, participants made a pleasantness judgment (pleasant = g, unpleasant = k) in the deep condition, and a line judgment (straight line = g, no straight line = k) in the shallow condition. In the extension condition, participants were asked: “Is the object in the circle larger or smaller than a shoebox in real life?” in the deep condition, and “Is the object shown in the circle larger or smaller than the shoebox on the screen?” in the shallow condition. They responded “bigger” by pressing “g” and “smaller” by pressing “k.” The recall phase, identical across all conditions, required participants to adjust the grayscale object’s color to match the encoded color. Feedback for the color setting was provided on a scale that indicated the degree of deviation between the participant’s adjusted color and the originally encoded color. This scale ranged from a happy emoji (low deviation) to an angry emoji (high deviation).

Each recall trial displayed an object from the previous encoding phase in grayscale within a grey circle. The circle was a color wheel with a random offset on each trial. Participants indicated the recalled color by moving their mouse on the color wheel, which adjusted the color of the displayed object. Participants could adjust the color until they are satisfied with the choice and click on a button submitting their response. After the feedback display (1s) the next trial started until all eight objects of the block were presented. Feedback was visually presented using a scale showing the absolute deviation between the original encoded color and the reported color. Greater deviations indicated poorer performance. At one end of the scale, a happy emoji represented perfect performance where the absolute color deviation was 0 degrees between the original and reported colors. In contrast, an angry emoji at the opposite end represented poor performance, with an absolute color deviation of 180 degrees. An example trial is depicted in Figure 1.

After completing 10 blocks in one encoding condition (e.g., deep), participants received an introduction to the other encoding condition (e.g., shallow). The procedure was the same (one practice block followed by 10 experimental blocks à 8 trials). After completing the experiments, participants completed the calibration procedure again and filled out a brief questionnaire. The questionnaire gathered information on demographics, task preference (“With which judgment task were you able to remember the color more easily?”), use of alternative encoding strategies (“Did you use a verbalization strategy for the memory task?”), and adherence to instructions (“Did you complete all tasks according to the instructions?”). Finally, participants were debriefed and thanked. The study lasted around one hour.

### Design and Analysis

The study design was a 2 × 2 mixed design with the between subjects variable instructions (replication vs. extension) and the within subject variable levels of processing (deep vs. shallow). The dependent variable was memory performance measured as the angular deviation from the target color in degrees. This value ranges from 0 to 180 with values closer to 0 indicating more precise recall. For each participant, we calculated the absolute median deviation per condition (i.e., response error). We chose to use the median (cf. pre-registration) rather than the mean for aggregating participant data because the median is less sensitive to outliers and skewed distributions and thus can be a better choice to represent central response tendencies. That said, we also ran the analysis with the mean and report it in the Supplement.

Data was prepared and analyzed in RStudio (Posit team, 2024) using R (R Core Team, 2023) with the packages *tidyverse* (Wickham et al., 2019), *ez* (Lawrence, 2016), *schoRsch* (Pfister & Janczyk, 2022), *apa* (Gromer, 2023), *apaTables* (Stanley, 2021). Stimulus materials, experiment programs, datafiles, and analysis scripts are on OSF (https://osf.io/5bzyt/).

## Results

**Figure 2:**
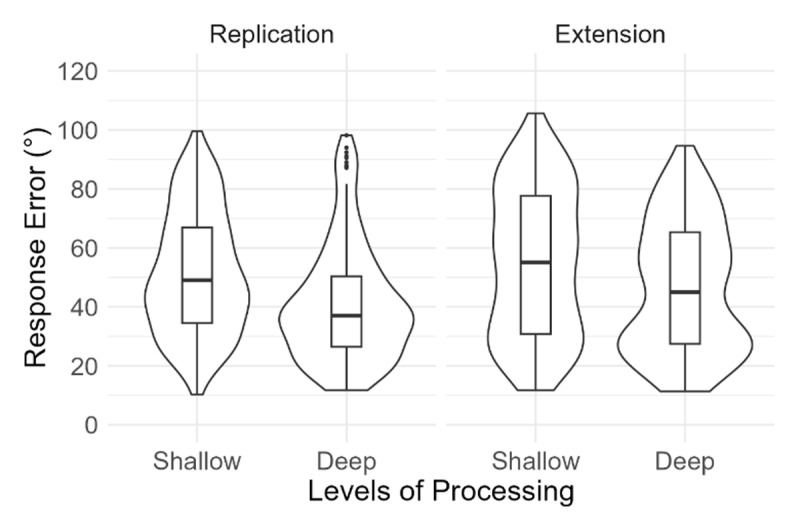
Response Error as a Function of Instructions (Replication, Extension) and Levels of Processing (Shallow, Deep). *Note*. Lower values represent better performance. The violin plots indicate the density of the data. The horizontal line inside each box plot indicates median performance (i.e., 50^th^ percentile). The lower and upper ends of each box represent the 25^th^ and 75^th^ percentiles, enclosing the interquartile range. The whiskers extend to 1.5 times the interquartile range from either end of the box. The points outside the whiskers of the boxplot are outliers. Spearman-Brown corrected reliability estimates with 95% confidence intervals were calculated for each condition using 5000 random splits with the R package *splithalf* (Parsons, 2021). Results indicated good reliability: Replication-shallow = 0.82 [0.78, 0.86], Replication-deep = 0.86 [0.83, 0.89], Extension-shallow = 0.88 [0.85, 0.91], Extension-deep = 0.87 [0.84, 0.90].

Absolute response errors as a function of instructions and levels of processing are depicted in Figure 2. The 2 × 2 ANOVA revealed a significant main effect of levels of processing, *F*(1, 305) = 103.18, *p* < .001, \[
\eta _p^2
\] = .25. Participants had better memory performance (i.e., lower response errors) in the deep (*M* = 43.7°, *SE* = 1.2°) than in the shallow (*M* = 53.0°, *SE* = 1.3°) encoding condition. The main effect of instruction was not significant, *F*(1, 305) = 3.39, *p* = .066, \[\eta _p^2\] = .01, and neither was the interaction, *F*(1, 305) = 0.79, *p* = .376, \[\eta _p^2\] < .01.

The different outlier exclusion criteria (cf. preregistration) revealed the same pattern of results except for the main effect of instruction, which became significant indicating better memory performance for the replication instruction (cf. Appendix). Critically, the main effect of levels of processing was significant and the interaction was nonsignificant.

To further corroborate our results, we conducted additional analyses which were not preregistered and are thus reported in the Supplement. First, we aggregated participant data using the mean. Although this analysis revealed an interaction between levels of processing and instruction, it is important to note, that the levels of processing effect was significant in both instruction conditions. The interaction indicates a larger effect in the replication instruction, suggesting that alternative explanatory factors may have contributed to the effect in the replication instruction. However, the small effect size suggests that the interaction is negligible. Further, we tested for potential block order effects. Critically, the results do not change the interpretation of the main analysis. For completeness, we also report the results of the encoding task in the Supplement. These analyses indicated comparable performance between the conditions.

### Posttest Questionnaire

In line with the behavioral results, the results of the posttest questionnaire revealed that the majority (178 participants) experienced the deep encoding condition as easier than the shallow condition (18 participants). The remaining 111 participants were indifferent. The majority (162 participants) indicated that they verbalized the color and the object during encoding, 91 participants indicated that they did not use any verbalization strategy, 36 participants indicated that they verbalized the color, and 18 participants indicated that they verbalized the object. See Table 1 for a detailed overview per condition.

**Table 1:** Number of Participants Indicating Experienced Task Easiness and Verbalization Strategy Use in the Posttest Questionnaire.


QUESTION	REPLICATION	EXTENSION

Task Easiness	*n*	*n*

deep	97 (61%)	81 (54%)

shallow	5 (3%)	13 (9%)

indifferent	56 (35%)	55 (37%)

Verbalization Strategy		

both	83 (53%)	79 (53%)

color	16 (10%)	20 (13%)

object	10 (6%)	8 (5%)

none	49 (31%)	42 (28%)


## Discussion

The present study replicated and extended previous findings on the levels of processing framework using nonverbal material with novel encoding instructions. In the replication instruction condition, participants either decided whether there was a straight line in the presented object (shallow encoding) or whether they liked the object (deep encoding). Given that pleasantness judgments can introduce alternative explanations for improved memory such as task-relevance, self-reference, and attentional scope, the primary aim of our study was to address these issues by using different encoding instructions (Bower & Karlin, 1974; Dubravac & Meier, 2023; Rogers et al., 1977). Our novel encoding instructions were designed to isolate the level of processing from these confounding factors, providing a clearer test of the levels of processing framework. In the extension instruction condition, participants compared the size of the presented object to the size of a displayed reference object (shallow encoding) or to the real-life size of the reference object (deep encoding).

Our results indicated better memory for deep compared to shallow encoding, suggesting that tasks requiring semantic processing (deep encoding) enhance memory performance more effectively than those requiring only perceptual processing (shallow encoding). Critically, this was observed across both the replication (pleasantness vs. straight-line judgment) and extension (real-life vs. displayed size judgment) instruction conditions, providing robust evidence for the applicability of the levels of processing framework to visual associative memory. This pattern remained consistent after applying different outlier exclusion criteria, suggesting a robust levels of processing effect that is not simply due to the greater relevance of color, higher self-reference, or broader attentional scope in the pleasantness judgment compared to the line judgment. Thus, our study supports previous related research (Baddeley & Hitch, 2017; Ovalle-Fresa, Uslu, et al., 2021).

When analyzing participant data aggregated with the mean, the interaction between levels of processing and instructions was significant, indicating a larger levels of processing effect for the replication condition compared to the extension condition (cf. Supplement). This result suggests that our new manipulation is a clearer test of the levels of processing framework. The key feature of our manipulation was that the display as well as the response dimension (larger vs. smaller) were kept the same across the deep and shallow encoding conditions. However, the effect size of the interaction was small, and the interaction did not reach significance when using the preregistered, more robust median-based aggregation method. This suggests that the interaction may be negligible. Taken together, this pattern of results raises the possibility that our new manipulation may not have entirely eliminated all alternative explanatory factors.

Specifically, self-reference could still play a role in the real-life size judgment task, as participants might imagine how they would use the objects in their own lives when considering their real-world sizes.^3^ Additionally, attentional scope may have differed between tasks: in the real-life size task, participants could focus solely on the target object since the reference object (the shoebox) remained constant and could be ignored. In contrast, the displayed size task required attention to both the target object and the varying size of the shoebox, potentially dividing attention and affecting encoding. Incorporating different reference objects (e.g., a fridge)^4^ could have compelled participants to attend to the reference object in both tasks, but selecting suitable objects compatible with our stimulus set is challenging. Although we cannot rule out all alternative explanations, our findings provide additional evidence supporting the generality of the levels-of-processing framework. Future studies should aim to further control for alternative factors to better understand the underlying mechanisms.

In line with Ovalle-Fresa, Uslu, et al. (2021), our posttest questionnaire indicated that most participants found the deep encoding condition easier than the shallow condition. These subjective reports align with our objective findings from the memory test, which showed that the deep encoding condition aids memory performance. Furthermore, our posttest questionnaire revealed that many participants reported using verbalization strategies, such as verbalizing the object and its color. This raises the question of whether the levels of processing effect truly applies to nonverbal material like pictures. As Ovalle-Fresa, Uslu, et al. found similar levels of processing effects with fractals, which are abstract, geometric shapes without verbal labels and limited distinctiveness (Ovalle-Fresa, Di Pietro, et al., 2021), we argue that the effects observed in our study may indeed extend to purely nonverbal material. However, future research should directly test other encoding instructions and their impact on memory for fractal-color associations. Furthermore, future research should explicitly control for or manipulate verbalization strategies to isolate their impact on memory performance. Another avenue for future research is to assess our novel encoding instructions for memory with different pictures and words to gain more insights into the processes involved in the levels of processing effect (D’agostino et al., 1977; Konstantinou & Gardiner, 2005; Meier et al., 2009; Nieznański, 2020).

In conclusion, our study provides evidence that the levels of processing framework applies to basic perceptual features in visual associative memory. By replicating previous findings and addressing potential confounding factors, we demonstrated that deeper encoding conditions lead to better memory performance than shallow encoding conditions. However, since alternative explanatory factors such as self-reference and attentional scope may still partly contribute to these effects, our results highlight the importance of semantic processing while also underscoring the need for further research to disentangle these factors in enhancing memory for visual information.

## Data Accessibility Statement

Stimulus materials, experiment programs, datafiles, and analysis scripts are on OSF (https://osf.io/5bzyt/).

## Additional File

The additional file for this article can be found as follows:

10.5334/joc.437.s1 Supplement. Additional analyses.

## Footnotes

This number differs from the preregistration because we planned to recruit 352 participants based on the number of students in the course but did not consider students who did not complete the course.

The color sampling procedure was as follows: The color wheel has 360 hues. As 360 divided by 80 gives 4.5, the distance between the colors was 4 or 5 in alternating order around the color wheel. Starting at 0°, we would continue with 4°, then 9°, 13°, 18°, 22°, 27°, 31°, 36°, 40°,…,355° (please note that 360° corresponds to the same hue as 0°). Please see the file “ExpList.csv” on OSF to see which hues were in which of the ten lists (see first two columns).

Thanks to an anonymous reviewer for pointing this out.

Thanks to Nuno D. Sobrinho and Alessandra Souza for this idea.

## Appendix

As specified in the preregistration we tested the robustness of our results by comparing results without outlier exclusion to the results after applying different outlier exclusion criteria to our main dependent variable (i.e., performance that differs +/–2 or +/–3 SD from the average group performance in a given condition or if participants lie outside the whiskers of the boxplot in a specific condition).

After applying the boxplot outlier exclusion criterion, we excluded nine participants of the replication instruction condition (cf. Fig. 2). Consistent with the ANOVA without outlier exclusions, the main effect of levels of processing was significant, *F*(1, 296) = 122.72, *p* < .001, \[\eta _p^2\] = .29. Participants had better memory performance (i.e., lower response errors) in the deep (*M* = 42.3°, *SE* = 1.1°) than in the shallow (*M* = 52.3°, *SE* = 1.3°) encoding condition. The interaction remained nonsignificant, *F*(1, 296) = 2.89, *p* = .090, \[\eta _p^2\] < .01. The main effect of instruction, however, became significant, *F*(1, 296) = 8.40, *p* = .004, \[\eta _p^2\] = .03. Participants in the replication instructions condition had better memory performance (*M* = 44.0°, *SE* = 1.1°) than participants in the extension instructions condition (*M* = 50.6°, *SE* = 1.4°).

From the original dataset we excluded 13 participants (two were from the extension instruction condition) that had higher response errors than two standard deviations from the group mean of a certain condition (no participant deviated –2 SD or +/- 3 SD from the group mean). Consistent with the ANOVA without outlier exclusions, the main effect of levels of processing was significant, *F*(1, 292) = 119.47, *p* < .001, \[\eta _p^2\] = .29. Participants had better memory performance (i.e., lower response errors) in the deep (*M* = 41.8°, *SE* = 1.1°) than in the shallow (*M* = 51.7°, *SE* = 1.3°) encoding condition. The interaction remained nonsignificant, *F*(1, 292) = 2.70, *p* = .101, \[\eta _p^2\] < .01. As with the boxplot outlier exclusion criterion, the main effect of instruction was significant, *F*(1, 292) = 8.72, *p* = .003, \[\eta _p^2\] = .03. Participants in the replication instructions condition had better memory performance (*M* = 43.5°, *SE* = 1.1°) than participants in the extension instructions condition (*M* = 50.0°, *SE* = 1.4°).

## References

[1] Baddeley, A. D., & Hitch, G. J. (2017). Is the Levels of Processing effect language-limited? Journal of Memory and Language, 92, 1–13. 10.1016/j.jml.2016.05.001

[2] Bower, G. H., & Karlin, M. B. (1974). Depth of processing pictures of faces and recognition memory. Journal of Experimental Psychology, 103(4), 751–757. 10.1037/h0037190

[3] Brodeur, M. B., Dionne-Dostie, E., Montreuil, T., & Lepage, M. (2010). The Bank of Standardized Stimuli (BOSS), a New Set of 480 Normative Photos of Objects to Be Used as Visual Stimuli in Cognitive Research. PLoS ONE, 5(5), e10773. 10.1371/journal.pone.0010773 20532245 PMC2879426

[4] Brysbaert, M. (2019). How many participants do we have to include in properly powered experiments? A tutorial of power analysis with reference tables. In Journal of Cognition (Vol. 2, Issue 1). Ubiquity Press. 10.5334/joc.72 PMC6640316 31517234

[5] Clark, J. H. (1924). The Ishihara Test for Color Blindness. American Journal of Physiological Optics, 5, 269–276.

[6] Craik, F. I. M., & Lockhart, R. S. (1972). Levels of processing: A framework for memory research. Journal of Verbal Learning and Verbal Behavior, 11(6), 671–684. 10.1016/S0022-5371(72)80001-X

[7] Craik, F. I. M., & Tulving, E. (1975). Depth of processing and the retention of words in episodic memory. Journal of Experimental Psychology: General, 104(3), 268–294. 10.1037/0096-3445.104.3.268

[8] D’agostino, P. R., O’neill, B. J., & Aivio, A. P. (1977). Memory for pictures and words as a function of level of processing: Depth or dual coding? In Memory & Cognition (Vol. 5, Issue 2). 10.3758/BF03197370 24202819

[9] Dubravac, M., & Meier, B. (2023). Cognitive load at encoding hurts memory selectivity. Quarterly Journal of Experimental Psychology, 76(7), 1515–1538. 10.1177/17470218221132846 36214174

[10] Gromer, D. (2023). apa: Format Outputs of Statistical Tests According to APA Guidelines. R package version 0.3.4. https://CRAN.R-project.org/package=apa

[11] Henninger, F., Shevchenko, Y., Mertens, U. K., Kieslich, P. J., & Hilbig, B. E. (2022). lab.js: A free, open, online study builder. Behavior Research Methods, 54(2), 556–573. 10.3758/s13428-019-01283-5 34322854 PMC9046347

[12] Konstantinou, I., & Gardiner, J. M. (2005). Conscious control and memory awareness when recognising famous faces. Memory, 13(5), 449–457. 10.1080/09658210444000016 16020375

[13] Lawrence, M. (2016). ez: Easy Analysis and Visualization of Factorial Experiments (version 4.4-0).

[14] Leding, J. K. (2018). The animacy advantage in memory: Manipulations of Levels of Processing and survival processing. The American Journal of Psychology, 131(3), 273–281. 10.5406/amerjpsyc.131.3.0273

[15] Levine, L. J., & Edelstein, R. S. (2009). Emotion and memory narrowing: A review and goal-relevance approach. Cognition & Emotion, 23(5), 833–875. 10.1080/02699930902738863

[16] Meier, B., Theiler-Bürgi, M., & Perrig, W. (2009). Levels of processing and amnesia affect perceptual priming in fragmented picture naming. International Journal of Neuroscience, 119(8), 1061–1075. 10.1080/00207450802336691 19922339

[17] Morris, C. D., Bransford, J. D., & Franks, J. J. (1977). Levels of processing versus transfer appropriate processing. Journal of Verbal Learning and Verbal Behavior, 16(5), 519–533. 10.1016/S0022-5371(77)80016-9

[18] Muhmenthaler, M. C., Dubravac, M., & Meier, B. (2023). How attention and knowledge modulate memory: The differential impact of cognitive conflicts on subsequent memory—A review of a decade of research. Frontiers in Cognition, 2. 10.3389/fcogn.2023.1125700

[19] Nieznański, M. (2020). Levels-of-processing effects on context and target recollection for words and pictures. Acta Psychologica, 209. 10.1016/j.actpsy.2020.103127 32603912

[20] Ooms, J. (2023). magick: Advanced Graphics and Image-Processing in R. In R package (R package version 4.8.1).

[21] Ovalle-Fresa, R., Di Pietro, S. V, Reber, T. P., Balbi, E., & Rothen, N. (2021). Standardized database of 400 complex abstract fractals. Behavior Research Methods, 54(5), 2302–2317. 10.3758/s13428-021-01726-y 34918225

[22] Ovalle-Fresa, R., Uslu, A. S., & Rothen, N. (2021). Levels of processing affect perceptual features in visual associative memory. Psychological Science, 32(2), 267–279. 10.1177/0956797620965519 33450171

[23] Parsons, S. (2021). splithalf: robust estimates of split half reliability. Journal of Open Source Software, 6(60), 3041. 10.21105/joss.03041

[24] Pfister, R., & Janczyk, M. (2022). schoRsch: Tools for Analyzing Factorial Experiments. R package version 1.10. https://CRAN.R-project.org/package=schoRsch

[25] Posit team. (2024). RStudio: Integrated Development Environment for R (2024.4.0.735). Posit Software, PBC.

[26] R Core Team. (2023). R: A language and environment for statistical computing. R Foundation for Statistical Computing. https://www.r-project.org/

[27] Rhodes, M. G., & Anastasi, J. S. (2000). The effects of a levels-of-processing manipulation on false recall. Psychonomic Bulletin & Review, 7(1), 158–162. 10.3758/BF03210735 10780030

[28] Richter, F. R., & Yeung, N. (2012). Memory and cognitive control in task switching. Psychological Science, 23(10), 1256–1263. 10.1177/0956797612444613 22972906

[29] Rogers, T. B., Kuiper, N. A., & Kirker, W. S. (1977). Self-reference and the encoding of personal information. Journal of Personality and Social Psychology, 35(9), 677–688. 10.1037/0022-3514.35.9.677 909043

[30] Sachdeva, C., Whelan, E., Ovalle-Fresa, R., Rey-Mermet, A., Ward, J., & Rothen, N. (2024). How perceptual ability shapes memory: An investigation in healthy special populations. Stage 1 Registered Report.

[31] Stanley, D. (2021). apaTables: Create American Psychological Association (APA) Style Tables (R package version 2.0.8).

[32] Symons, C. S., & Johnson, B. T. (1997). The self-reference effect in memory: A meta-analysis. Psychological Bulletin, 121(3), 371–394. 10.1037/0033-2909.121.3.371 9136641

[33] Wickham, H., Averick, M., Bryan, J., Chang, W., McGowan, L., François, R., Grolemund, G., Hayes, A., Henry, L., Hester, J., Kuhn, M., Pedersen, T., Miller, E., Bache, S., Müller, K., Ooms, J., Robinson, D., Seidel, D., Spinu, V., … Yutani, H. (2019). Welcome to the Tidyverse. Journal of Open Source Software, 4(43), 1686. 10.21105/joss.01686

