# Supplementary material for: Levels of Processing Effects on Memory for Color-Object Associations
Source: J Cogn. 2025 Mar 4;8(1):25. doi: 10.5334/joc.437 (PMC11887462; doi:10.5334/joc.437)

Supplement

Mean-based Aggregation

We run the same analysis as in the main manuscript but instead of medians we now use means to aggregate participant data. Absolute response errors as a function of instructions and levels of processing are depicted in Figure 1. The 2x2 ANOVA revealed a significant main effect of levels of processing, $F(1, 305) = 133.05$, $p < .001$, $\eta_p^2 = .30$. The main effect of instruction was non-significant, $F(1, 305) = 2.15$, $p = .144$, $\eta_p^2 < .01$. The interaction was significant, $F(1, 305) = 4.52$, $p = .034$, $\eta_p^2 = .01$. Importantly, the levels of processing effect was significant in both the replication, $t(157) = 9.44$, $p < .001$, 95% CI [7.05, 10.78], and extension instruction condition, $t(148) = 6.85$, $p < .001$, 95% CI [4.37, 7.91].

Figure 1

Response Error as a Function of Instructions (Replication, Extension) and Levels of Processing (Shallow, Deep)

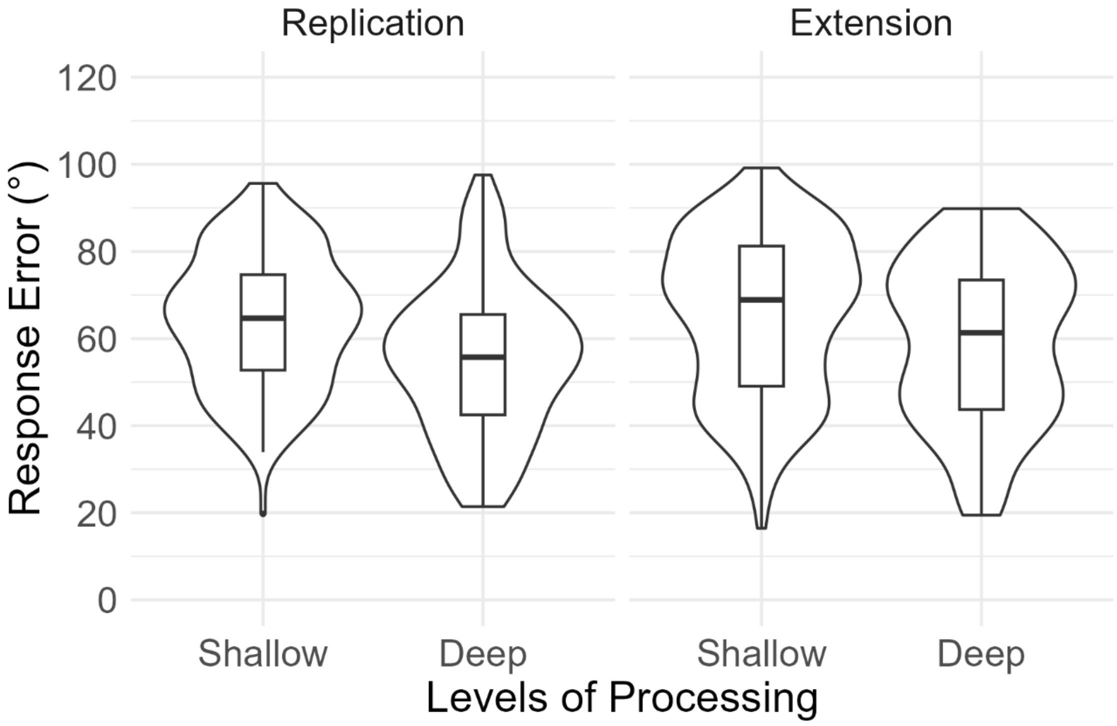

To evaluate whether the levels of processing effect differed across instruction conditions, we calculated a difference score for each participant by subtracting the deep condition value from the shallow condition value. These difference scores were then subjected to an independent samples t-test comparing the levels of processing effect between the replication and extension conditions. The analysis revealed a significant difference between the two groups, $t(304.85) = 2.13$, $p = .034$, 95% [0.21, 5.34]. The mean difference score was larger in the replication condition ($M = 8.9°$, $SE = 0.9°$) than in the extension condition ($M = 6.1°$, $SE = 0.9°$), suggesting that the levels of processing effect was more pronounced in the replication condition. This result should be interpreted with caution, as the effect size of the interaction is small, and the same analysis using the median-based aggregation method reported in the main manuscript did not reach significance.

Block Order Effects

To test potential block order effects, we added the factor “order” with the levels DS (deep followed by shallow, n = 150) and SD (shallow followed by deep, n = 157) to our main factors; levels of processing (deep, shallow) and instruction (replication, extension). The 2x2x2 ANOVA revealed a significant main effect of levels of processing, $F(1, 303) = 118.81$, $p < .001$, $\eta_p^2 = .28$. The main effect of instruction was not significant, $F(1, 303) = 3.64$, $p = .057$, $\eta_p^2 = .01$, and neither was the main effect of order, $F(1, 303) = 0.74$, $p = .392$, $\eta_p^2 < .01$.

The interaction between instruction and order was significant, $F(1, 303) = 4.07$, $p = .044$, $\eta_p^2 = .01$. In the DS order, performance was better in the replication instruction group ($M = 42.8°$, $SE = 1.62°$) than in the extension instruction group ($M = 51.9°$, $SE = 1.93°$), $t(288.26) = -3.61$, $p < .001$, 95% CI [-14.07, -4.15]. In the SD order, performance did not differ, $t(296.34) = 0.10$, $p = .922$, 95% CI [-4.83, 5.34].

The interaction between levels of processing and order was also significant, $F(1, 303) = 39.21$, $p < .001$, $\eta_p^2 = .11$, suggesting a stronger levels of processing effect in the DS order. Most importantly, the

levels of processing effect was significant in both order types. In the DS order, performance was better in the deep (*M* = 39.9°, *SE* = 1.47°) than shallow (*M* = 54.7°, *SE* = 1.92°) encoding condition, *t*(149) = -11.69, *p* < .001, 95% CI [-17.34, -12.33]. Similarly, in the SD order, performance was better in the deep (*M* = 47.4°, *SE* = 1.85°) than shallow (*M* = 51.4°, *SE* = 1.76°) encoding condition, *t*(156) = -3.45, *p* < .001, 95% CI [-6.35, -1.73].

The interaction between instruction and levels of processing was not significant, *F*(1, 303) = 1.08, *p* = .298, η_p^2 < .01, and neither was the three-way interaction, *F*(1, 303) = 0.03, *p* = .868, η_p^2 < .01. Figure 2 illustrates the results.

Figure 2

Response Error as a Function of Instructions (Replication, Extension), Levels of Processing (Shallow, Deep), and Block Order (DS = deep-shallow, SD = shallow-deep)

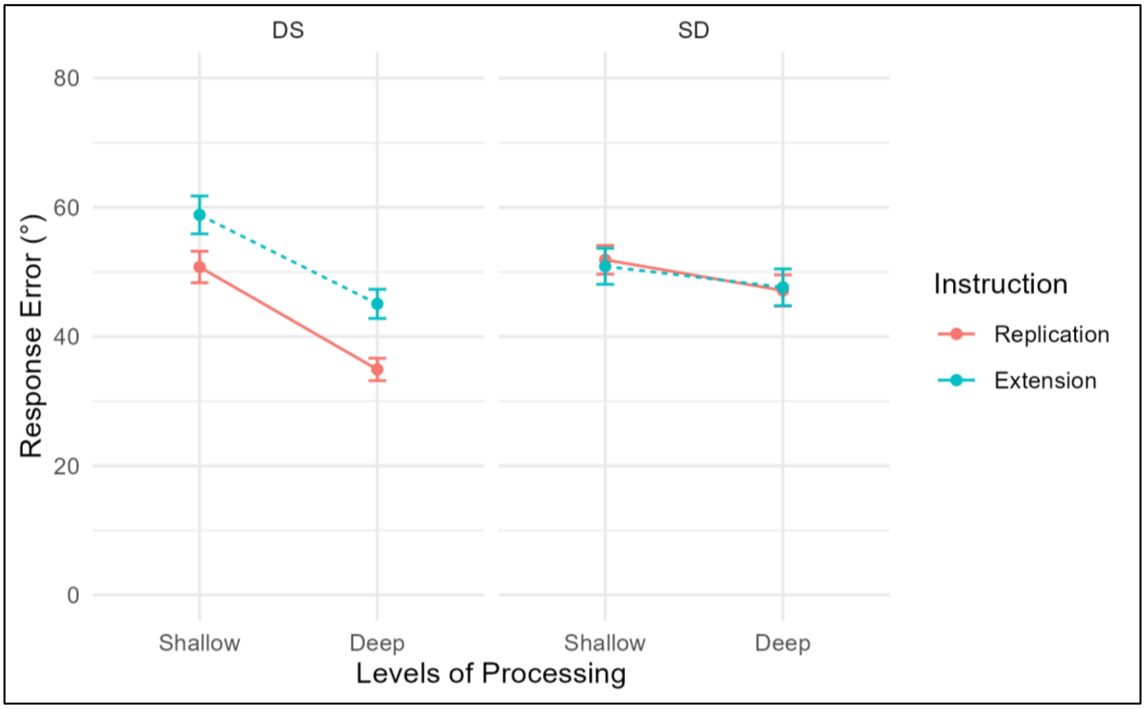

Encoding Task

As there was a time limit for the response in the encoding task, we analyzed the number of missing responses. The deep and shallow blocks each had 80 trials. In the replication, the mean number of missing responses was 22 (*SE* = 2) in the deep, and 20 (*SE* = 2) in the shallow condition. In the extension, the mean number of missing responses was 19 (*SE* = 2) in the deep, and 18 (*SE* = 2) in the shallow condition.

Next, we analyzed response times in the encoding task. We excluded ten participants (7 extension, 3 replication) because they did not respond within the time limit. We aggregated participants response times by calculating the median per condition. The 2x2 ANOVA indicated no significant main effect of instruction, $F(1, 298) = 0.36$, $p = .548$, $\eta_p^2 < .01$, nor levels of processing $F(1, 298) = 3.85$, $p = .051$, $\eta_p^2 = .01$. The interaction was also non-significant, $F(1, 298) = 1.47$, $p = .226$, $\eta_p^2 < .01$.

Running the same ANOVA with the means of participants response times per condition, resulted in a significant main effect of levels of processing, $F(1, 298) = 7.32$, $p = .007$, $\eta_p^2 = .02$. Participants responded faster in the deep (*M* = 484 ms, *SE* = 14 ms) than the shallow encoding condition (*M* = 513 ms, *SE* = 14 ms). The small effect size indicates a negligible effect. The other main effect was non-significant, $F(1, 298) = 0.34$, $p = .561$, $\eta_p^2 < .01$. The interaction was also non-significant, $F(1, 298) = 0.40$, $p = .527$, $\eta_p^2 < .01$. Figure 3 illustrates the results with medians and Figure 4 illustrates the results with means.

Figure 3

Median Response Times

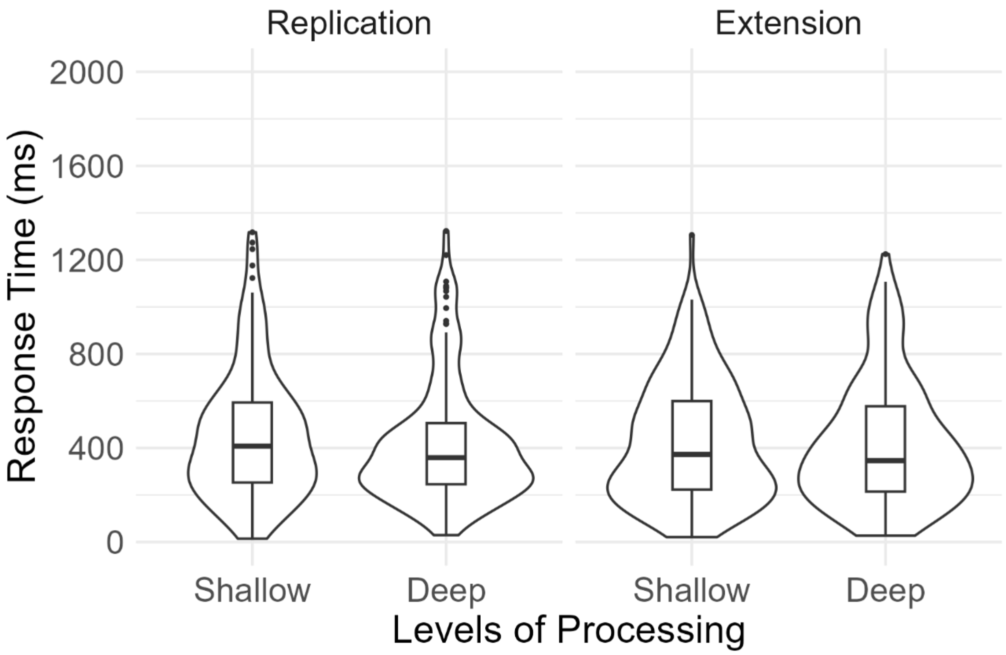

Figure 4

Mean Response Times

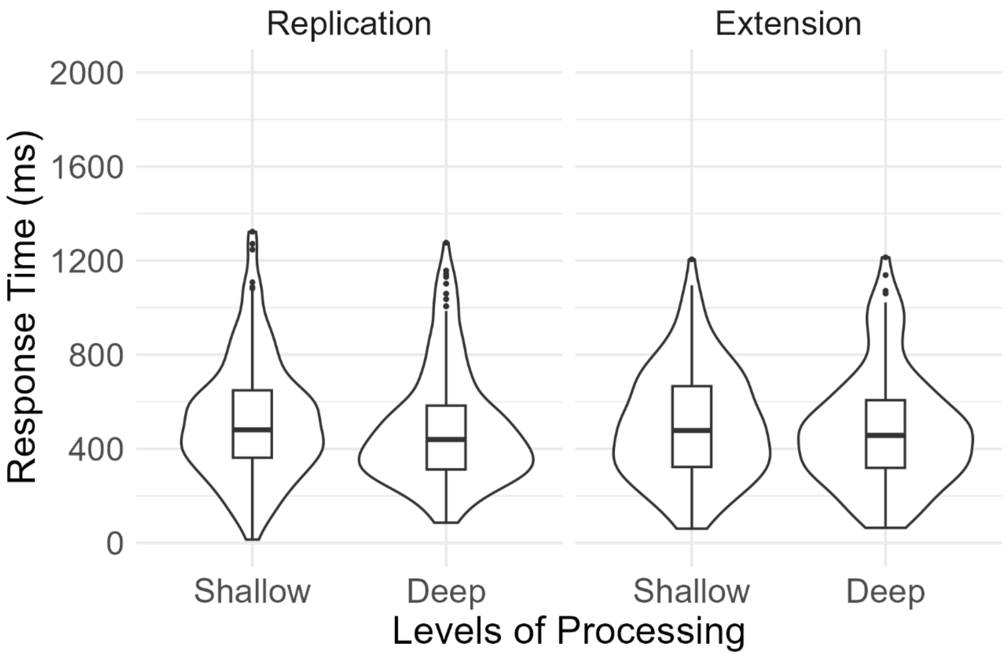

Supplement: Supplement. — Additional analyses. [file joc-8-1-437-s1.pdf]
